# Association of serum vitamin D levels on *Helicobacter pylori* infection: a retrospective study with real-world data

**DOI:** 10.1186/s12876-023-03037-2

**Published:** 2023-11-13

**Authors:** Dan Liu, Li Ren, Dapeng Zhong, Wei Zhang, Wen Wen Li, Jie Liu, Chuan Han

**Affiliations:** 1 Department of Endocrinology, General Hospital of the Western Theater Command, No 270, Tianhui Road, Chengdu, Sichuan Province 610036 China; 2 State Key Laboratory of Cancer Biology, National Clinical Research Center for Digestive Diseases, Xijing Hospital of Digestive Diseases, Air Force Military Medical University, Xi’an, Shaanxi Province 710000 China

**Keywords:** *Helicobacter pylori*, Infection, Vitamin D, Education levels, Family size, Annual income

## Abstract

**Objective:**

The aim of this study was to determine whether serum vitamin D levels are associated with *H. pylori* infection and whether low serum vitamin D levels are an independent risk factor for *H. pylori* infection.

**Methods:**

We conducted a retrospective analysis of a multicenter cohort study from 2017 to 2019. A total of 415 *H. pylori*^*+*^ patients and 257 *H. pylori*^−^ patients aged between 18 and 75 years with both 13 C-urea breath test and serum vitamin D level results were included from four hospitals. A questionnaire was used to collect information on potential factors influencing *H. pylori* infection.

**Results:**

Serum vitamin D levels were significantly lower in the *H. pylori*^*+*^ group than in the *H. pylori*^*−*^ group (16.7 ± 6.6 ng/ml vs. 19.2 ± 8.0 ng/ml, p < 0.05). Using a cutoff value of 20 ng/ml, the *H. pylori* infection rate was significantly higher in the vitamin D-deficient group (< 20 ng/ml) than in the vitamin D-nondeficiency group (≥ 20 ng/ml) (66.5% vs. 51.0%, p < 0.001). Ordered logistic regression analysis showed that serum vitamin D levels < 20 ng/ml (OR: 1.652, 95% CI: 1.160–2.351, p = 0.005), higher education levels (OR: 1.774, 95% CI: 1.483–2.119, p < 0.001), family size ≥ 4 (OR: 1.516, 95% CI: 1.081–2.123, p = 0.016), and lower annual income (OR: 1.508, 95% CI: 1.289–1.766, p < 0.001) were independent risk factors for *H. pylori* infection.

**Conclusion:**

Lower serum vitamin D levels may be associated with an increased risk of *H. pylori* infection, and lower serum vitamin D levels are an independent risk factor for increasing *H. pylori* infection rates. Randomized controlled trials are needed to determine whether supplementation with vitamin D can reduce *H. pylori* infection rates.

## Introduction

*Helicobacter pylori* (*H. pylori*) infection is currently the most important controllable risk factor for gastric cancer, with 90% of noncardia gastric cancers being associated with *H. pylori* infection [1–5]. China is one of the countries with a high prevalence of *H. pylori* infection, with an overall infection rate of 56.22%. The infection rate of *H. pylori* in Tibet is as high as 84.62%, ranking first in the world [6]. There are variations and trends in *H. pylori* infection rates among different regions in China. Using the carbon-urea breath test as the diagnostic method for current infection, the lowest current infection rate was found in Guangdong Province at 42%, while the highest was in Shaanxi Province at 64%. Therefore, preventing *H. pylori* infection is crucial for reducing the incidence of gastric cancer in China.

Vitamin D is a micronutrient that regulates bone metabolism. However, several studies have found that vitamin D3 decomposition product 1 (VDP1) can induce cell membrane collapse, leading to the lysis of *H. pylori* cells [7, 8]. Additionally, vitamin D3 can reactivate the acidification and degradation function of autolysosomes through the activation of the PDIA3-STAT3-MCOLN3-Ca^2+^ axis, thereby eliminating the survival of *H. pylori* hidden in the autophagy of cells [9]. Vitamin D3 can also induce the VDR-CAMP signaling pathway to eradicate *H. pylori* in the stomach [10]. Furthermore, it can protect gastric mucosal epithelial cells against *H. pylori* infection-induced apoptosis through the VDR-dependent c-Raf/MEK/ERK pathway [11].

However, there is currently controversy regarding the relationship between serum vitamin D levels and *H. pylori* infection in clinical studies. In a cross-sectional study conducted in 2007 on end-stage renal disease patients undergoing maintenance dialysis, a significant positive correlation was found between serum vitamin D levels and serum *H. pylori*^*−*^ specific IgG antibody titers (r = 0.36, P = 0.043) [12]. This suggests that vitamin D analogs may have antibacterial effects against *H. pylori*. Another study conducted in Japan on healthy elderly women aged 70–99 years in nursing homes found that the prevalence of *H. pylori* infection was significantly lower in subjects receiving vitamin D3 supplementation than in unheated individuals (p < 0.05), indicating a suppressive effect of long-term vitamin D3 intake on *H. pylori* infection [13]. Antonio Antico et al. also found that serum vitamin D levels were significantly lower in *H. pylori*^*−*^ related gastric inflammation patients than in healthy individuals, suggesting that individuals with lower serum vitamin D levels may be more susceptible to *H. pylori* infection [14]. A clinical study conducted in Italy found that the proportion of vitamin D deficiency in the *H. pylori*^+^ group was significantly higher than that in the *H. pylori*^*−*^ group (86% vs. 67.3%, P = 0.014) [15]. A cross-sectional study of 294 patients who visited a hospital in Lebanon in 2016 for dietary habits and *H. pylori* infection found that participants with a university degree or higher education (OR = 2.74; CI = 1.17–6.44), patients with a history of peptic ulcer disease (OR = 3.80; CI = 1.80–8.01), stomach adenocarcinoma (OR = 3.99; CI = 1.35–11.83), and those with vitamin D levels below normal (OR = 29.14; CI = 11.77–72.13) had a higher risk of *H. pylori* infection [16]. A cross-sectional study of individuals aged 65 and older found that the proportion of patients with *H. pylori*^+^ and vitamin D deficiency (< 20 ng/mL) was higher than that of the *H. pylori*^*−*^ group (86% vs. 67.3%, p = 0.014). The proportion of *H. pylori*^+^ patients decreased with increasing serum vitamin D levels (p = 0.010) [17]. A large cross-sectional study conducted in infants and young children found that the prevalence of vitamin D deficiency in the *H. pylori*^+^ and *H. pylori*^*−*^ groups was 20.7% and 12.1%, (P < 0.001) [18]. A study of a large electronic database of medical records from the Israeli population’s health maintenance organization found a negative correlation between serum vitamin D levels and *H. pylori* infection (p < 0.001). The odds of *H. pylori* detection being positive were 31% higher in patients with serum vitamin D levels < 20 ng/mL than in those with levels ≥ 20 ng/mL (OR 1.31, 99% CI 1.22–1.4, p < 0.001) [19]. However, a community-based study from Taiwan, which included 1126 *H. pylori*^+^ and 987 *H. pylori*^*−*^ patients, found no significant difference in the average serum vitamin D levels between the two groups. Further stratification by age also did not reveal any differences [20].

Due to the limited and controversial clinical studies on the relationship between serum vitamin D levels and *H. pylori* infection, we conducted a retrospective analysis of a cohort study to determine the impact of serum vitamin D levels on *H. pylori* infection and whether low serum vitamin D levels independently increase the risk of *H. pylori* infection.

## Data and methods

### Study subjects

We conducted a retrospective analysis of case data from a multicenter cohort study conducted between 2017 and 2019. The study included a total of 496 *H. pylori −* positive (*H. pylori*^+^) and 257 *H. pylori −* negative (*H. pylori*^−^) patients between the ages of 18 and 75 who had 13 C-urea breath test results and serum levels of vitamin D measured using chemiluminescence assay. The study was conducted at Xijing Hospital of Air Force Medical University, Xianyang Central Hospital, The First Affiliated Hospital of Zhengzhou University, and West Zone General Hospital. Information on *H. pylori*^+^ and *H. pylori*^−^ patients was collected using a questionnaire on the factors affecting *H. pylori* infection. Exclusion criteria included the following: (a) age less than 18 years old or over 75 years old; (b) previous history of *H. pylori* eradication therapy; (c) previous history of gastric surgery; (d) pregnancy or lactation; (e) major systemic diseases (e.g., hypertension, diabetes, hypothyroidism, metabolic syndrome, adrenal insufficiency, inflammatory bowel disease, systemic lupus erythematosus, rheumatoid arthritis, systemic vasculitis, HIV/AIDS, etc.); (f) diseases affecting serum vitamin D levels (e.g., hyperthyroidism, malabsorption, rickets, bone tumors, Cushing’s syndrome, severe liver disease with Child‒Pugh class B or C, renal failure with serum creatinine > 177 µmol/L, alcoholism); (g) use of antibiotics, acid-suppressing drugs or bismuth preparations within the past 8 weeks; (h) daily supplementation of vitamin D; and (i) incomplete case data. Finally, a total of 415 *H. pylori*^+^ and 257 *H. pylori*^−^ patients were included.

### Statistical analysis

Statistical analysis was conducted using SPSS version 22.0 software (IBM, Armonk, NY, USA). Double-entry and validation were used to ensure the accuracy of the data collected from the cohort study. The normality of continuous variables was tested using the Kolmogorov‒Smirnov test, and nonnormally distributed variables are presented as medians (range), while normally distributed variables are presented as the means ± SDs. Group comparisons were performed using t tests, Wilcoxon rank-sum tests, or chi-square tests as appropriate. Two-tailed P < 0.05 was considered statistically significant. Univariate analysis was used with *H. pylori* infection as the dependent variable and various factors as independent variables, and ordered logistic regression was used to determine if lower levels of serum vitamin D were independent risk factors for increased *H. pylori* infection risk. The strength of association was measured using odds ratios (ORs) and 95% confidence intervals (95% CIs).

## Results

### Patient characteristics

As shown in Table 1, information on *H. pylori*^+^ and *H. pylori*^−^ patients was collected using a questionnaire on the factors affecting *H. pylori* infection, including sex, age, occupation, residential area, body type, marital status, education level, family size, annual income, cigarette smoking, alcohol consumption, history of periodontal disease, hygiene of dining place, main source of drinking water, drinking unheated water, and vitamin D serum level. The hygiene of dining place was self-assessed by the patients using the questionnaire as either clean and hygienic or relatively poor. The main source of drinking water for patients was assessed using the questionnaire. If it came from purified water or tap water, it was defined as clean. If it came from well water, river water, or spring water, it was defined as probably polluted.

**Table 1:** Univariate analysis of factors influencing *H. pylori* infection

Variables	*H. pylori*-positive (N = 415)	*H. pylori*-negative (N = 257)	P value
Sex, n (male/female)	194/221	127/130	0.501
Age, y (mean ± SD)	47.3 ± 12.8	48.5 ± 10.1	0.163
Occupation, n (indoor/outdoor worker)	116/299	79/178	0.439
Residential area, n (urban/rural)	316/99	181/76	0.101
Body type, n(normal/overweight/obesity)	289/112/14	168/70/19	0.059
Marital status, n (married/single)	376/39	250/7	0.001
Education level, n (elementary school and below/middle school/university and above)	48/188/179	64/125/68	< 0.001
Family size, n (< 4/≥4 people)	167/248	125/132	0.033
Annual income, n (low/medium low/medium/medium high/high)	28/93/112/84/98	2/41/68/68/78	< 0.001
Cigarette smoking, n (yes/no)	88/327	69/188	0.093
Alcohol consumption, n (yes/no)	32/383	21/236	0.830
History of periodontal disease, n (yes/no)	119/296	73/184	0.940
Hygiene of dining place, n (good/poor)	321/94	187/70	0.179
Main source of drinking water, n (clean/probably polluted)	363/52	212/45	0.074
Drinking unheated water, n (yes/no)	93/322	66/191	0.332
Vitamin D serum level, n (< 20/≥20 ng/ml)	311/104	157/100	< 0.001

Note: Annual income, low, < 5000; medium low, 5001–20,000; medium, 20,001–50,000; medium high, 50,001–80,000; high, ≥ 80,000 CNY.

**Fig. 1:**
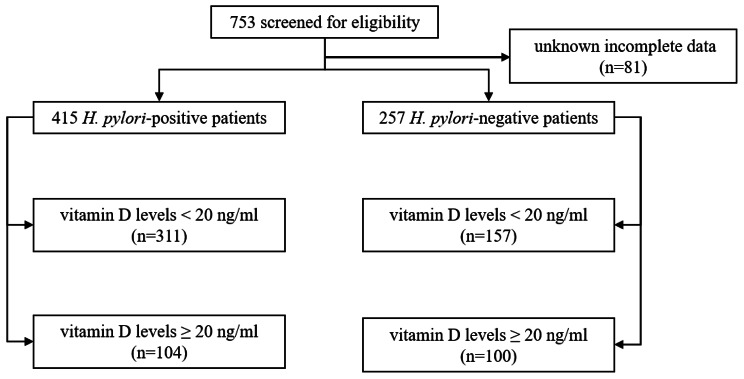
Flowchart of participant selection and grouping

As shown in Fig. 1, there were a total of 496 *H. pylori*^+^ and 257 *H. pylori*^*−*^ patients in the cohort. After excluding 81 *H. pylori*^+^ patients with incomplete data, a total of 415 *H. pylori*^+^ and 257 *H. pylori*^*−*^ patients were included in the analysis. Comparing the serum vitamin D levels between the two groups, the *H. pylori*^+^ group had significantly lower levels than the *H. pylori*^*−*^ group (16.7 ± 6.6 ng/ml vs. 19.2 ± 8.0 ng/ml, p < 0.001), as shown in Table 2.

**Table 2:** Differences in serum vitamin D levels between the *H. pylori*^+^ and *H. pylori*^−^ groups

Variables	*H. pylori*-positive (N = 415)	*H. pylori*-negative (N = 257)	T value(P value)
Vitamin D (25[OH]D), ng/ml (mean ± SD)	16.7 ± 6.6	19.2 ± 8.0	4.117(<0.001)

### Difference in *H. pylori* infection rate between patients with serum vitamin D levels < 20 ng/ml and ≥ 20 ng/ml

The patients were divided into a vitamin D deficiency group (< 20 ng/ml) and a nondeficiency group (≥ 20 ng/ml) according to a cutoff value of 20 ng/ml for serum vitamin D levels. The difference in the *H. pylori* infection rate between the two groups was compared, as shown in Table 3. The vitamin D deficiency group had a significantly higher *H. pylori* infection rate than the nondeficiency group (66.5% vs. 51.0%, p < 0.001), suggesting a possible association between vitamin D deficiency and a higher *H. pylori* infection rate.

**Table 3:** Difference in the *H. pylori* infection rate between patients with serum vitamin D levels < 20 ng/ml and ≥ 20 ng/ml

Vitamin D (25[OH]D)	*H. pylori*-positive (N = 415)	*H. pylori*-negative (N = 257)	Infection rate (%)	*P* value
<20 ng/ml	311	157	66.5%	-
≥ 20 ng/ml	104	100	51.0%	< 0.001

### Univariate analysis of factors influencing the *H. pylori* Infection rate

Univariate analysis was conducted on all factors collected using a questionnaire on the factors affecting *H. pylori* infection in both the *H. pylori*^+^ and *H. pylori*^*−*^ groups. As shown in Table 1, there were no significant differences in sex, age, occupation, residential area, body type, cigarette smoking, alcohol consumption, history of periodontal disease, hygiene of dining place, main source of drinking water and drinking unheated water between the *H. pylori*^+^ and *H. pylori*^*−*^ groups. However, there were significant differences in marital status (married vs. single, 60.1% vs. 84.8%, p = 0.001), education level (elementary school and below vs. middle school vs. university and above, 42.9% vs. 60.1% vs. 72.5%, p < 0.001), family size (< 4 vs. ≥ 4, 57.2% vs. 65.3%, p = 0.033), annual income (low vs. medium low vs. medium vs. medium high vs. high, 93.3% vs. 69.4% vs. 62.2% vs. 55.3% vs. 55.7%, p < 0.001), and serum vitamin D levels (< 20 ng/ml vs. ≥ 20 ng/ml, 66.5% vs. 51.0%, p < 0.001) between the two groups.

### Multivariate analysis of factors influencing *H. pylori* Infection

As shown in Table 4, ordered logistic regression analysis was conducted on the factors with significant differences in the univariate analysis. The results revealed that serum vitamin D level < 20 ng/ml (OR: 1.652, 95% CI: 1.160–2.351, p = 0.005), higher education level (OR: 1.774, 95% CI: 1.483–2.119, p < 0.001), family size ≥ 4 (OR: 1.516, 95% CI: 1.081–2.123, p = 0.016), and lower annual income (OR: 1.508, 95% CI: 1.289–1.766, p < 0.001) were independent risk factors influencing *H. pylori* infection. Additionally, married was not found to be an independent protective factor for *H. pylori* infection (OR: 0.430, 95% CI: 0.182–1.012, p = 0.053).

**Table 4:** Multivariate analysis of factors influencing *H. pylori* infection

Variables	OR(95%CI)	P value
Vitamin D serum level (< 20/≥20 ng/mL)	1.652(1.160–2.351)	0.005
Marital status (married/single)	0.430(0.182–1.012)	0.053
Education level (university and above/middle school/elementary school and below)	1.774(1.483–2.119)	< 0.001
Family size (≥ 4/< 4 people)	1.516(1.081–2.123)	0.016
Annual income, n (low/medium low/medium/medium high/high)	1.508(1.289–1.766)	< 0.001

## Discussion

*H. pylori* infection is a significant risk factor for the development of gastric cancer in the majority of patients [1]. *H. pylori* infection accounts for 90% of noncardia gastric cancer cases [2]. Therefore, active prevention and treatment of *H. pylori* infection are essential for reducing the incidence of gastric cancer. Vitamin D is an important micronutrient that affects bone metabolism. However, basic research has demonstrated that vitamin D3 metabolites can induce the collapse of *H. pylori* cell membranes and eliminate *H. pylori* by activating multiple signaling pathways [7–11]. Serval clinical research has also shown that Vitamin D influences *H. pylori* infection and eradication [12–25].

Due to conflicting results on the association between serum vitamin D levels and *H. pylori* infection and the lack of large sample, multicenter, and multivariable case‒control studies, we designed this study to further explore whether serum vitamin D levels are an independent risk factor for *H. pylori* infection. Our study found that the *H. pylori*^+^ group had significantly lower serum vitamin D levels than the *H. pylori*^*−*^ group (16.7 ± 6.6 ng/ml vs. 19.2 ± 8.0 ng/ml, p < 0.001). Defining vitamin D deficiency as serum vitamin D levels < 20 ng/ml, the prevalence of *H. pylori* infection was significantly higher in the vitamin D-deficient group than in the nondeficient group (66.5% vs. 51.0%, p < 0.001). Furthermore, ordered logistic regression analysis showed that serum vitamin D levels < 20 ng/ml (OR: 1.652, 95% CI: 1.160–2.351, p = 0.005), higher education level (OR: 1.774, 95% CI: 1.483–2.119, p < 0.001), larger family size (≥ 4 members) (OR: 1.516, 95% CI: 1.081–2.123, p = 0.016), and lower annual income (OR: 1.508, 95% CI: 1.289–1.766, p < 0.001) were independent risk factors for *H. pylori* infection. This suggests that lower serum vitamin D levels may be associated with an increased risk of *H. pylori* infection, and lower levels of serum vitamin D are independent risk factors for increased *H. pylori* infection rates. Interestingly, our study, along with another study, indicated that higher education levels were associated with higher rates of *H. pylori* infection. This could be due to higher education levels leading to a greater likelihood of undergoing *H. pylori* testing, resulting in relatively higher rates of *H. pylori* infection being detected. Although our sample size was relatively large, the lack of specific data on the impact of vitamin D on *H. pylori* infection rates hindered our ability to calculate the exact sample size. Additionally, our study did not include vitamin D supplementation; therefore, further evaluation in larger randomized controlled trials is required to assess whether vitamin D supplementation can reduce the rate of *H. pylori* infection.

## Data Availability

The datasets used and analyzed during the current study are available from the corresponding author upon reasonable request.
